# Supplementary material for: Causal Relationship between Aging and Anorexia Nervosa: A White-Matter-Microstructure-Mediated Mendelian Randomization Analysis
Source: Biomedicines. 2024 Aug 16;12(8):1874. doi: 10.3390/biomedicines12081874 (PMC11351342; doi:10.3390/biomedicines12081874)
Supplement: Supplementary file 1 [file biomedicines-12-01874-s001.zip › Supplementary Figure/Figure S4/Singe SNP plot of MR analysis for TL to ubm-b-1562.pdf]

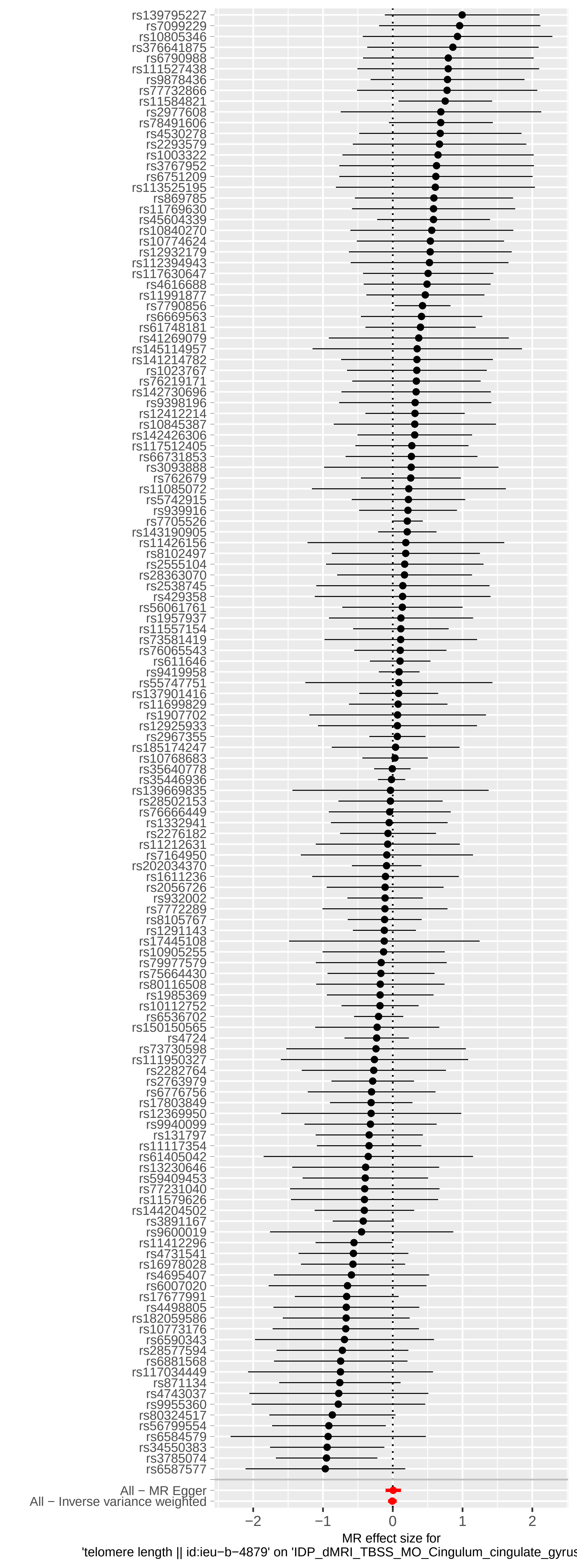
rs139795227
rs7099229
rs10805346
rs376641875
rs6790988
rs111527438
rs9878436
rs77732866
rs11584821
rs2977608
rs78491606
rs4530278
rs2293579
rs1003322
rs3767952
rs6751209
rs113525195
rs869785
rs11769630
rs45604339
rs10840270
rs10774624
rs12932179
rs112394943
rs117630647
rs4616688
rs11991877
rs7790856
rs6669563
rs61748181
rs41269079
rs145114957
rs141214782
rs1023767
rs76219171
rs142730696
rs9398196
rs12412214
rs10845387
rs142426306
rs117512405
rs66731853
rs3093888
rs762679
rs11085072
rs5742915
rs939916
rs7705526
rs143190905
rs11426156
rs8102497
rs2555104
rs28363070
rs2538745
rs429358
rs56061761
rs1957937
rs11557154
rs73581419
rs76065543
rs611646
rs9419958
rs55747751
rs137901416
rs11699829
rs1907702
rs12925933
rs2967355
rs185174247
rs10768683
rs35640778
rs35446936
rs139669835
rs28502153
rs76666449
rs1332941
rs2276182
rs11212631
rs7164950
rs202034370
rs1611236
rs2056726
rs932002
rs7772289
rs8105767
rs1291143
rs17445108
rs10905255
rs79977579
rs75664430
rs80116508
rs1985369
rs10112752
rs6536702
rs150150565
rs4724
rs73730598
rs111950327
rs2282764
rs2763979
rs6776756
rs17803849
rs12369950
rs9940099
rs131797
rs11117354
rs61405042
rs13230646
rs59409453
rs77231040
rs11579626
rs144204502
rs3891167
rs9600019
rs11412296
rs4731541
rs16978028
rs4695407
rs6007020
rs17677991
rs4498805
rs182059586
rs10773176
rs6590343
rs28577594
rs6881568
rs117034449
rs871134
rs4743037
rs9955360
rs80324517
rs56799554
rs6584579
rs34550383
rs3785074
rs6587577
All − MR Egger
All − Inverse variance weighted
−2
−1
0
1
2
MR effect size for
'telomere length || id:ieu−b−4879' on 'IDP_dMRI_TBSS_MO_Cingulum_cingulate_gyrus
